# Pulpotomy of Symptomatic Permanent Teeth with Carious Exposure Using Mineral Trioxide Aggregate

**Published:** 2013-05-01

**Authors:** Imad Hassan Barngkgei, Esam Saleh Halboub, Roula Safouh Alboni

**Affiliations:** 1 Faculty of Dentistry, Damascus University, Damascus, Syria; 2 Oral Medicine Department, Faculty of Dentistry, Damascus University. Damascus, Syria/ Oral Medicine and Periodontology Department, Faculty of Dentistry, Sana`a University, Sana`a, Yemen; 3 Department of Operative Dentistry and Endodontics, Faculty of Dentistry, Damascus University, Damascus, Syria

**Keywords:** Dental Pulp Exposure, Endodontic, Mineral Trioxide Aggregate, Permanent Dentition, Pulpotomy, Root Canal Therapy

## Abstract

**Introduction:**

To evaluate the clinical and radiographic outcomes of pulpotomy treatment with mineral trioxide aggregate (MTA) in symptomatic mature permanent teeth with carious exposure.

**Materials and Methods:**

Ten patients aged 27-54 years presented with 11 symptomatic permanent teeth (n=11). Each offending carious tooth was clinically and radiographically determined. We removed caries as conservatively as possible; however pulp exposure was inevitable. ProRoot MTA pulpotomy was performed on these teeth. The patients were followed-up clinically and radiographically for 24-42 months.

**Results:**

Immediate relief of patients` symptoms occurred. Moreover, teeth responses to the electric pulp tester were within normal range on follow-up appointment and the radiographs did not reveal any abnormality/lesion in the periapical areas.

**Conclusion:**

Pulpotomy using MTA could be a good alternative for root canal therapy (RCT) for managing symptomatic mature permanent teeth with carious exposure, however further large-scale multicenter clinical trials are highly encouraged to confirm this hypothesis.

## 1. Introduction

Vital pulp therapies aim to maintain tooth vitality, and functionality and to render the tooth asymptomatic (1). Pulpotomy, for instance, is a procedure in which the coronal pulp that presumed to be inflamed and/or infected is surgically removed, and the remaining radicular dental pulp is covered at the orifices with a suitable material that protects the pulp from further injury and permits and promotes healing (2, 3). Traditionally, the term "pulpotomy" has implied removal of the pulpal tissue to the cervical line, to be differentiated from “Cvek type” pulpotomy or “partial pulpotomy” in which just a portion of the coronal pulp is removed (4). Indications of pulpotomy include primary teeth with irreversible coronal pulpitis or exposed vital pulps. Pulpotomy is considered as a treatment for immature permanent teeth with pulp exposure due to caries or trauma that gives evidence of extensive coronal pulpitis, and also as an emergency procedure for permanent mature teeth until root canal treatment can be accomplished (2).

Disagreement exists concerning pulp capping and pulpotomy as a permanent treatment option in mature permanent teeth. It is universally accepted; however, that vital pulp therapy is indicated for pulp exposure in teeth with incompletely formed roots. But once root formation has been completed, root canal therapy (RCT) should be performed (2). The best root canal filling is the healthy pulp tissue, and it should not be assumed that every damaged pulp must be extirpated and that pulp conservation is not satisfactory procedure (5). However, pulp exposure due to deep caries, in particular, normally results in the mature tooth requiring RCT. It is the only procedure which insures removal of the bacteria that invaded the pulp (6).

MTA has attracted attention since its introduction to the field of endodontics (7). It has a lot of advantages compared to other conventional endodontic materials; *e.g.* its excellent sealing ability (8), biocompatibility (9), dentin bridge formation in cases of pulp capping (10, 11) and pulpotomy (12) in addition to inducing proliferation of the pulpal cells (13). Although many studies showed that MTA is the material of choice for vital pulp therapy in primary and young permanent teeth, (14-17) other studies found no significant differences (18, 19).

Studies regarding the management of carious exposure in mature (fully developed) teeth are scare and conflicting. Accordingly, the aim of this clinical trial was to evaluate the clinical and radiographic outcomes of mineral trioxide aggregate (MTA) pulpotomy as permanent treatment of symptomatic mature permanent teeth with carious exposure.

## 2. Material and Methods

**Figure 1.**
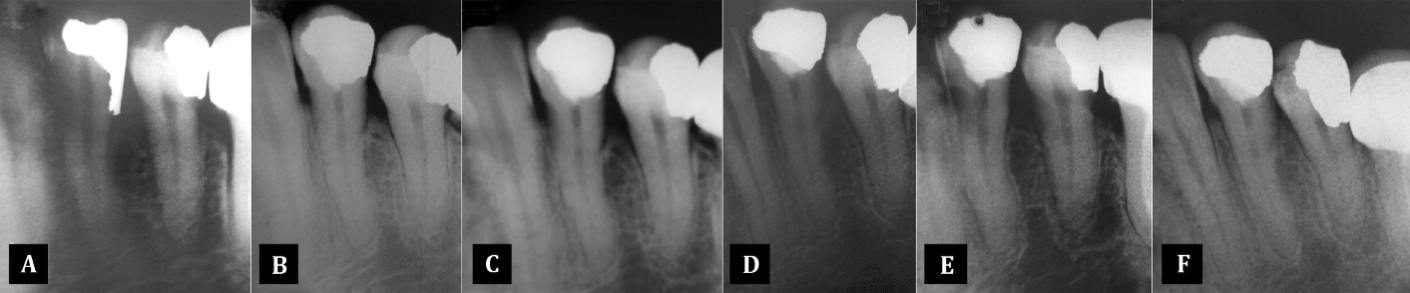
Periapical diagnostic and follow-up radiographs of tooth 34; A) diagnostic radiograph showing a secondary caries under amalgam restoration; B) 3 month after MTA pulpotomy; the periapical structures appear normal and; C-F) normal periapical structures upon radiographic follow up visits at 12, 24, 36 and 42 months, respectively

**Figure 2.**
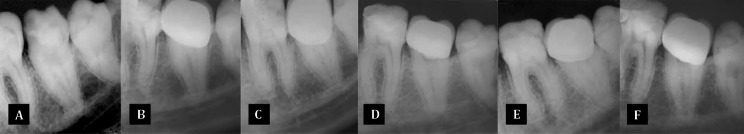
Periapical diagnostic and follow up radiographs of tooth 37: A) diagnostic radiograph showing a secondary caries under a composite restoration; B) 3 months after MTA pulpotomy radiograph after crowning the tooth; the periapical structure appears normal and; C-F) normal periapical structures upon radiographic follow-up visits at 12, 24, 36 and 42 months, respectively

This study was conducted on the records of 10 patients (4 males and 6 females; aged 17-54 years) that referred to Operative Dentistry and Endodontics clinic of Damascus Dental School between 2007 and 2008 due to stimulated intense pain on sweets and/or cold food/drinks. On clinical examinations, the offending teeth (n=11) were either deeply carious or had large restoration. Stimulation with electric pulp tester (CIR Anthos, Imola, Italy) resulted in a more intense response in the offending teeth compared to the adjacent and opposite teeth. These clinical symptoms were consistent with reversible pulpitis. In addition, diagnosis of apical periodontitis was excluded by percussion palpation examination. Moreover radiographs revealed “normal periapical structures” *i.e.* score 1 according to Ørstavik periapical index (PAI) (20) (Figures 1A, 2A). The patients were informed regarding the pulpotomy treatment using MTA in case the pulp exposure happened. The department board adopted this procedure as an alternative to the RCT in such cases. Each patient signed an informed consent.

Following local anesthesia with 2% lidocaine 1:80000 epinephrine (Kwang Myung, Korea) and, when possible, rubber dam isolation, caries/restoration was removed using a high speed air motor with water coolant. Low speed air motor with water coolant was then used carefully to remove the carious dentin. Teeth were included only if carious exposure and pulp bleeding were evident upon excavation; this was the most important inclusion criterion for case series. The roof of the pulp chamber along with coronal pulpal tissue was removed using high speed air motor under water coolant. The bleeding was controlled using a small piece of sterile cotton pellet for about 5 minutes. MTA (ProRoot; Dentsply, Tulsa, OK, USA) was mixed according to the manufacturer`s instructions and was applied on the floor of the pulp chamber with a thickness of approximately 2-3 mm and root canal orifices; then a wet cotton pellet was placed on the layer of MTA. Temporary filling (Litark, Lascod, Italy) was applied till the next appointment in 2 days.

In the next appointment, the patients reported slight discomfort which was gradually diminishing and cold and/or sweet food/drinks did not initiate pain anymore. MTA was checked to confirm setting using a small rounded burnisher. Then, accordingly, a permanent filling of polycarboxylate cement (Spofa Dental, Adhesor® carbofine, Republic Czech) and amalgam restoration (GK amalgam capsule, China) was employed. Two teeth were indicated for crown restoration due to the weakness of the remaining structure. They were crowned 1 month and 1 year post-pulpotomy respectively (Table 1).

**Table 1.** Distribution of the 11 permanent mature teeth that were cariously exposed and treated by pulpotomy using MTA

Patient`s Number	Age	Gender	Tooth number	Pretreatment status	Follow-up period	Vitality response
**1**	20	Female	46	Deep Caries	28	Yes
**2**	20	Female	45	Deep Caries	24	Yes
**3**	23	Male	44	Deep Caries	24	Yes
**4**	17	Male	24	Deep Caries	26	Yes
**5**	17	Male	25	Deep Caries	28	Yes
**6**	26	Male	28	Deep Caries	30	Yes
**7**	26	Male	16	Deep Caries	24	N/A (crown)
**8**	54	Female	31	Deep Caries	26	Yes
**9**	38	Female	34	Large Restoration; Secondary Caries	42	Yes
**10**	42	Female	36	Deep Caries	42	Yes
**11**	24	Female	37	Large Restoration; Secondary Caries	42	N/A (crown)

The patients were re-evaluated after 1, 3 and 6 months, then every six months. Signs and symptoms including pain, swelling, sinus tract, tenderness to percussion were examined clinically while evidence of periapical or furcal pathosis or root resorption were evaluated with periapical radiographs (Figures 1B-1F, 2B-2F). All patients were asked whether they had experienced spontaneous pain, pain on chewing or pain stimulated by hot/cold. Electric pulp testing was applied, where feasible, in all follow-up visits.

## 3. Results

The mean age of the patient was 29 years; ranged from 17 to 54 year and the mean follow-up period was 30.5 months; ranged from 24-42 months (Table 1).

Clinical examination during the follow-up periods revealed that all patients were asymptomatic and free of clinical signs/symptoms including pain, swelling, presence of sinus tract, and tenderness to percussion. Electric pulp testing in the follow-up visits where applicable, measured higher scores for all treated teeth compared to the opposite or neighboring teeth. All the patients were asymptomatic at one month, 3-months and 6-months and then every 6 months follow-ups (6-9 follow-up visits). The electric pulp testing, when applicable, revealed delayed positive response of all tested teeth in the all follow-up visits; the electric pulp testing could not be performed on the two teeth that had been crowned (Table 1). However, during crown preparation, local anesthesia was used because of the accompanied pain. All treatment was deemed successful at time due to absence of clinical and radiographic signs.

No changes in the periapical status of the treated teeth were evident on the all Follow-up periapical radiographies (Figures 1B-1F, 2B-2F).

## 4. Discussion

Managing curiously exposed pulp in adults has been a controversial issue. Although RCT is indicated for managing symptomatic mature permanent teeth with carious exposure (2); it has a well-accepted prognosis (21). However, it is expensive, complex and time-consuming treatment (22, 23). Moreover, RCT is not a complicated-free procedure. Unusual canal shape (*e.g.* c-shaped canals), dilaceration, unusual number of canals or calcification in the root canal strongly complicate the endodontic treatment (24). RCT imposes many consequences such as making the tooth non-vital, removing of substantial amount of tooth structure, and therefore brittleness of the remaining structure. Consequently, an economic, conservative, and simple technique would be desirable (22). A recent systematic review concluded that vital pulp therapy should be considered as an alternative treatment (25). Overall, the success of vital pulp therapy techniques in curiously exposed teeth is dependent upon the technique Employed, the inflammatory status of the pulp tissue, the period of observation, the criteria used to determine the success and the type (biocompatibility) of pulp therapy agent used (26).

Successful outcomes have been demonstrated for CH partial pulpotomy in curiously exposed young permanent teeth. Dentin bridging and pulp health were more predictable with MTA pulpotomy (18, 27). However, there are as yet the only two following studies evaluated the outcome of MTA pulpotomy in symptomatic mature permanent teeth with carious exposure.

Eghbal *et al*. studied the short term clinical and histological outcomes of MTA pulpotomy of 12 mature permanent molars with irreversible pulpitis (28). Clinically, no complaint of discomfort or tenderness in the next day was reported. The teeth were extracted two months post treatment for histological study where dentinal bridge was evident in all cases. Witherspoon *et al*. (29) on the other hand, revealed good clinical and radiographic outcomes of MTA pulpotomy for 19 symptomatic permanent teeth in 14 patients. Although, the mean follow-up period was somewhat long (19.7 months), only 4 teeth were mature since the patients were young (age ranged 7-16 years).

The influence of the patient’s age on the treatment outcome is a matter of controversy. In contrast to Aguilar and Linsuwanont (25), Bjorndal *et al*. observed that younger patients were associated with a higher successful rate of vital pulp therapy (30).

Symptomatic mature teeth with deep caries are ordinarily treated with indirect pulp capping unless the adopted conservative caries removal leads to pulp exposure where RCT is indicated (22, 31-33). During the period between 2007 and 2008, hundreds of symptomatic deeply-carious mature teeth were treated accordingly. However, 10 patients, with curiously exposed teeth agreed (informed consent) to be treated with MTA pulpotomy. In the current study, the success observed with MTA vital pulp therapy opens new horizons in endodontics; the 24-42 months outcome revealed the efficiency of MTA pulpotomy in preserving the vitality of the curiously exposed symptomatic mature permanent teeth while relieving the patient’s symptoms.

## 5. Conclusion

In conclusion, pulpotomy using MTA could be a good alternative for RCT for managing symptomatic mature permanent teeth with carious exposure. Since samples treated in this study were limited in number, large-scale multicenter clinical trials are highly encouraged.

## References

[1] Caliskan MK (1995). Pulpotomy of carious vital teeth with periapical involvement.. Int Endod J..

[2] Endodontists AAo. (2004). Guide to Clinical Endodontics..

[3] Farooq NS, Coll JA, Kuwabara A, Shelton P (2000). Success rates of formocresol pulpotomy and indirect pulp therapy in the treatment of deep dentinal caries in primary teeth.. Pediatr Dent..

[4] Cvek M (1978). A clinical report on partial pulpotomy and capping with calcium hydroxide in permanent incisors with complicated crown fracture.. J Endod..

[5] Pitt Ford TR, Pitt Ford TR (2004). Dental Pulp.. Harty’s Endodontics in Clinical Practice..

[6] Taintor JF, Biesterfeld RC, Langeland K (1981). Irritational or reparative dentin. A challenge of nomenclature.. Oral Surg Oral Med Oral Pathol..

[7] Eidelman E, Holan G, Fuks AB (2001). Mineral trioxide aggregate vs. formocresol in pulpotomized primary molars: a preliminary report.. Pediatr Dent..

[8] Tsatsas DV MH, Kerezoudis NP (2005). Sealing effectiveness of materials used in furcation perforation in vitro.. Int Dent J..

[9] Torabinejad M, Parirokh M (2010). Mineral trioxide aggregate: a comprehensive literature review--part II: leakage and biocompatibility investigations.. J Endod..

[10] Ford T, Torabinejad M, Abedi H, Bakland L, Kariyawasam S (1996). Using mineral trioxide aggregate as a pulp-capping material.. J Am Dent Assoc..

[11] Parolia A, Kundabala M, Rao NN, Acharya SR, Agrawal P, Mohan M, Thomas M (2010). A comparative histological analysis of human pulp following direct pulp capping with Propolis, mineral trioxide aggregate and Dycal.. Aust Dent J..

[12] Faraco IM Jr, Holland R (2001). Response of the pulp of dogs to capping with mineral trioxide aggregate or a calcium hydroxide cement.. Dent Traumatol..

[13] Fridland M, Rosado R (2005). MTA solubility: a long term study.. J Endod..

[14] Karabucak B, Li D, Lim J, Iqbal M (2005). Vital pulp therapy with mineral trioxide aggregate.. Dent Traumatol..

[15] Nair PN, Duncan HF, Pitt Ford TR, Luder HU (2008). Histological, ultrastructural and quantitative investigations on the response of healthy human pulps to experimental capping with mineral trioxide aggregate: a randomized controlled trial.. Int Endod J..

[16] Al-Hiyasat AS, Barrieshi-Nusair KM, Al-Omari MA (2006). The radiographic outcomes of direct pulp-capping procedures performed by dental students: a retrospective study.. J Am Dent Assoc..

[17] Accorinte MLR, Holland R, Reis A, Bortoluzzi MC, Murata SS, Dezan E, Souza V, Alessandro LD (2008). Evaluation of mineral trioxide aggregate and calcium hydroxide cement as pulp-capping agents in human teeth.. J Endod..

[18] Qudeimat MA, Barrieshi-Nusair KM, Owais AI (2007). Calcium hydroxide vs mineral trioxide aggregates for partial pulpotomy of permanent molars with deep caries.. Eur Arch Paediatr Dent..

[19] Tuna D, Olmez A (2008). Clinical long-term evaluation of MTA as a direct pulp capping material in primary teeth.. Int Endod J..

[20] Orstavik D (1996). Time-course and risk analyses of the development and healing of chronic apical periodontitis in man.. Int Endod J..

[21] Kojima K, Inamoto K, Nagamatsu K, Hara A, Nakata K, Morita I, Nakagaki H, Nakamura H (2004). Success rate of endodontic treatment of teeth with vital and nonvital pulps. A meta-analysis.. Oral Surg Oral Med Oral Pathol Oral Radiol Endod..

[22] Asgary S, Eghbal MJ, Ghoddusi J, Yazdani S (2013). One-year results of vital pulp therapy in permanent molars with irreversible pulpitis: an ongoing multicenter, randomized, non-inferiority clinical trial.. Clin Oral Investig..

[23] Bender I (2000). Reversible and irreversible painful pulpitides: diagnosis and treatment.. Aust Endod J..

[24] Dorn SO, Gartner AH, Cohen S BR (1994). Case Selection and Treatment Planning.. Pathways of the Pulp..

[25] Aguilar P, Linsuwanont P (2011). Vital pulp therapy in vital permanent teeth with cariously exposed pulp: a systematic review.. J Endod..

[26] Waterhouse PJ NJ, Whitworth JM, Soames JV (2000). Primary molar pulp therapy--histological evaluation of failure.. Int J Paediatr Dent..

[27] Committee CA. (2011). Pulp Therapy Subcommittee (2009) Guideline on Pulp Therapy for Primary and Immature Permanent Teeth..

[28] Eghbal MJ, Asgary S, Baglue RA, Parirokh M, Ghoddusi J (2009). MTA pulpotomy of human permanent molars with irreversible pulpitis.. Aust Endod J..

[29] Witherspoon DE, Small JC, Harris GZ (2006). Mineral trioxide aggregate pulpotomies: a case series outcomes assessment.. J Am Dent Assoc..

[30] Bjorndal L, Reit C, Bruun G, Markvart M, Kjaeldgaard M, Nasman P, Thordrup M, Dige I, Nyvad B, Fransson H, Lager A, Ericson D, Petersson K, Olsson J, Santimano EM, Wennstrom A, Winkel P, Gluud C (2010). Treatment of deep caries lesions in adults: randomized clinical trials comparing stepwise vs. direct complete excavation, and direct pulp capping vs. partial pulpotomy.. Eur J Oral Sci..

[31] Pitt Ford TR, Rhodes JS, Pitt Ford HE (2002). Endodontics: problem-solving in clinical practice..

[32] Amini P, Parirokh M (2008). The importance of long time follow-up after vital pulp therapy: A case report.. Iran Endod J..

[33] Asgary S, Eghbal MJ, Ghoddusi J (2007). SEM evaluation of pulp reaction to different pulp capping materials in dog’s teeth.. Iran Endod J..

